# Surgical healing beyond the scalpel: exploring the impact of depressive symptoms on functional recovery in total knee arthroplasty patients

**DOI:** 10.1186/s13018-023-04302-6

**Published:** 2023-11-04

**Authors:** Harshith Neelaraju, Mahesh Gangaiah, Prabhat Mittal

**Affiliations:** Department of Orthopaedics, Sapthagiri Institute of Medical Sciences and Research Centre, Bengaluru, India

**Keywords:** Depression, Total knee arthroplasty

## Abstract

**Background:**

Numerous recent studies have explored the association between the mental health condition of patients before surgery and the outcomes of total knee arthroplasty. The objective of this study was to determine the prevalence of depressive symptoms among individuals undergoing total knee arthroplasty and to investigate the impact of pre-operative depressive symptoms as a significant and independent predictor on various health-related quality of life measures for patients undergoing knee surgery.

**Material and methods:**

During the period spanning from August 2019 to May 2020, an orthopedic database was established for the purpose of assessing patients' conditions before their surgeries. The data collection process occurred at three distinct intervals: prior to the surgery, as well as at the third and sixth months following the surgical procedure. In this study, we undertook an evaluation of both pre-operative and postoperative depressive symptoms, as well as functional status, utilizing various self-report measures. These measures included the Becks Depression Scale, the Western Ontario and McMaster Universities Osteoarthritis Index, and the Knee Society Clinical Rating System.

**Results:**

A total of 150 patients were included in the study. The proportion of patients who were severely distressed decreased from 99% (149) at the baseline assessment to 76% (114) who had mild depression and 24% (36) at borderline at 3-months of follow-up. At 6-month follow-up period, 85% (128) patients were classified as normal, with 15% (22) displaying mild distress levels.

**Conclusions:**

Patients experiencing depression exhibited notable enhancements in various outcome measures. The findings from this study underscore a two-way relationship between mental health and surgical outcomes. Specifically, the surgical intervention yielded significant improvements in mental health status. Conversely, poorer pre-operative mental health status emerged as a predictive factor for comparatively less favorable outcomes stemming from the surgery.

**Supplementary Information:**

The online version contains supplementary material available at 10.1186/s13018-023-04302-6.

## Introduction

Osteoarthritis ranks as the second most prevalent rheumatologic condition and holds the distinction of being the most common joint disease in India, with an incidence ranging from 22 to 29% [1]. Females are usually more generally affected than men with greater age. The pervasiveness of knee surgical procedures has been consistently expanding each year as life expectancy increases [2]. The rate of complications has been assessed to be 4.7% which incites higher financial burden, lesser recuperation time with lower personal life quality [3].

Depression is estimated to be 29.9% prevalent and is the most typical mood disorder which has an adverse outcome postoperatively on total knee replacements [4]. Identifying these pre-operative risk factors is essential before surgery. Intervention, stratification, and optimization of post–operative outcomes are essential, else, resulting in decreased functional improvement, prolonged rehabilitation, and extended dissatisfaction on patients' behalf [5]. The National Institutes of Health have created PROMIS (Patient-Reported Outcomes Measurement Information System) to provide an assessment of patient-reported outcomes which has proven to be simple, effective, and able to generalize over an array of clinical patients [6]. These terms provide useful outlined data about health and are useful predictive indicators of healthcare utilization and future mortality [6–8].

Numerous studies have revealed that pre-operative mental distress is predictive of various outcome measures, including pain, function, satisfaction, and quality of life, in patients undergoing total knee arthroplasty[9–18]. However, it is important to note that several of these studies have been constrained by relatively small sample sizes [13].

The core objective of this study is to delve into the occurrence of symptoms related to depression among individuals undergoing total knee arthroplasty. As a secondary aim, the research aims to assess how pre-operative depressive symptoms function as a noteworthy and independent predictor, influencing various health-related quality of life (HRQOL) measurements, including PROMIS scores, within the context of knee surgery patients.

## Materials and methods

This research was conducted in the form of an observational cross sectional study. To facilitate this, an orthopedic database was established and employed to assess patients' conditions prior to surgery, spanning from August 2019 to May 2020. All patients meeting the stipulated inclusion criteria and undergoing knee surgery at our institution were considered eligible participants, provided they granted informed consent. The collection of study data took place on three occasions: the day before surgery, 3 months post-surgery, and 6 months post-surgery.

The inclusion criteria encompassed the following: (1) Individuals undergoing primary total knee arthroplasty, and (2) individuals aged over 12 years. Those excluded from the study consisted of those undergoing emergency surgery, revisions of prior total joint prostheses, or individuals grappling with conditions like malignancy, infection, or neurological diseases.

The demographic information, including the presence of depressive symptoms, was self-reported by each patient as a part of the data collection process.

Patients participating in the study underwent assessment based on:

1. BECKS DEPRESSION SCALE (for assessing depressive symptoms) [19]
2. WOMAC AND KSS SCORE (Domains: Physical Function, Pain Interference, Fatigue, Social Satisfaction, and Depressive symptoms) [20–23].

The Beck Depression Inventory (BDI) which is also referred to as the Beck Depression Scale is a utilized questionnaire that individuals complete, on their own. Its purpose is to evaluate the intensity of symptoms, in people. The Beck Depression Inventory (BDI) is a questionnaire that aims to evaluate the presence of symptoms. It consists of 21 multiple choice questions, each describing behaviors and feelings associated with depression. Respondents assess their experiences, over the two weeks by choosing the option that best fits their situation. Each response is given a score on a scale of 0 to 3, where 0 represents the absence of the symptom and 3 indicates its presence. To determine the severity of depression all question scores are added together resulting in a score ranging from 0 to 63. Interpreting these scores a range of 0–13 suggests no depression while scores between 14 and 19 indicate depression. A score from 20 to 28 suggests depression and anything between 29 and 63 points, toward depression.

Every patient slated for Total Knee Replacement (TKR) surgery underwent a comprehensive evaluation encompassing general health status and knee-specific assessments using the WOMAC scale upon admission. In the context of this analysis, health-related measurements, demographic factors, past medical history, and prevailing medication details were extracted from the patients' medical records. The assessment of postoperative functional progress was accomplished during an outpatient department (OPD) visit or via telephonic communication conducted by a research assistant. Subsequently, the collected data were diligently inputted into the study's database for further analysis.

## Psychosocial and functional outcome measures

We conducted an assessment of patients' pre-operative and postoperative depressive symptoms and functional status using various self-report measures, including the Beck's Depression Scale, Western Ontario and McMaster Universities Osteoarthritis Index (WOMAC).

The WOMAC serves as a tool to capture perceived capabilities in individuals afflicted by knee and hip joint arthritis. Consequently, it has evolved into a standard approach for appraising the outcomes of total hip and knee arthroplasty procedures. Comprising three dimensions, it gauges the patient's perception of pain, joint stiffness, and overall function. For our study, we employed the WOMAC total score, along with its physical function, pain, and stiffness dimensions, as dependent measures [20–22].

Lastly, we conducted an analysis of the range of motion data both before and after the surgery for all patients to ascertain whether objective functional measurements exhibited disparities. Our assessment encompassed specific measurements, including passive and active flexion and extension.

## Analysis

The study employed t tests and χ2 analyses to uncover potential differences in outcomes based on demographic and clinical variables. The sample was divided into individuals who had undergone surgery and met the inclusion criteria, resulting in a group of *n* = 150. Analysis of covariance (ANCOVA) was utilized to compare subjects' scores on various psychosocial and functional measures at both baseline and follow-up, while adjusting for significant disparities in demographic factors.

Paired sample t tests were employed to ascertain noteworthy differences in outcome measure scores before and after surgery. For clarity, all follow-up WOMAC-related scores were subjected to a natural logarithmic transformation as using logarithms can be beneficial, in handling data stabilizing variances and establishing relationships. This approach helps improve the accuracy and validity of analyses. However, data presented in tables retained their original measurement scale for easier interpretation.

The data underwent stepwise regression analysis to discern whether demographic attributes, clinical features, or baseline Knee Society Score (KSS) subscale scores could predict WOMAC pain, stiffness, and functional outcomes following TKA. Age at follow-up and gender were the primary demographic variables included in the model. These factors, along with clinical characteristics and baseline KSS, were introduced separately as distinct blocks. Functional outcomes were quantified using the natural logarithm of WOMAC subscale scores during the follow-up period. KSS function scale (*P* < 0.001), WOMAC scale (*P* < 0.001), and Beck's Depression scale (*P* < 0.0001) was considered statistically significant.

## Results

### Demographics and clinical variables

Of the 150 subjects with Beck’s Depression Scale, 149 (99.3%) were classified as moderate to severely depressed. Table 1 displays the demographic data of the patients. Out of the total study population, 113 patients had at least one comorbid illness. 63 percent of patients among males and females had a cardiovascular disease diagnosis, 31% among males and 38% of female patients had an endocrine problem, and 4% of males and 2% of female patients had pulmonary conditions (Additional file 1).

**Table 1:** Baseline characteristics

Characteristics	Males *n*	%	Females *n*	%
Total Patients (*N* = 150)	47	31.3	103	68.6
State:	44	93.6	89	86.4
Telangana Andhra Pradesh	3	6.3	10	9.7
Other state	0	0	4	3.8
*Comorbidity*				
HTN	27	57.4	59	57.2
T2DM	13	27.6	26	25.2
CAD	3	6.3	7	6.7
Asthma	4	8.5	2	1.9
Thyroid Disorder	2	4.2	13	12.6
Age, years (Mean ± SD) *	Mean	SD	Mean	SD
	65.8	8.4	59.4	7.3

Values are expressed in percentage (%) for categorical variables and for continuous variable value is expressed in Mean + /_ SD

^*^P value is calculated using Pearson’s unpaired t test with p < 0.05 as significant

## Psychosocial and functional outcomes- interpretation

Before undergoing surgery, subjects who experienced psychological distress displayed notably lower scores across various psychosocial and functional metrics, except for the WOMAC pain scale. However, post-surgery, a significant improvement was observed in these areas (refer to Table 2). Notably, the KSS function scale (*P* < 0.001), WOMAC scale (*P* < 0.001), and Beck's Depression scale (*P* < 0.0001) remained relatively lower compared to their pre-operative status.

**Table 2:** Analysis of depression levels. Beck's depression scores improved significantly in the post-operative period compared to the pre-operative levels

Becks Depression Scale	Pre-op (%)	Post-op 3 months (%)	Post-op 6 months (%)
Normal (1–10)	0	0	85%
Mild (11–16)	0	76%	15%
Borderline (17–20)	0.6%	24%	0
Moderate (21–30)	89.3%	0	0
Severe (31–40)	10%	0	0
Extreme (> 40)	0	0	0

When examining change scores, postoperative improvement was significantly greater across multiple measures, including the KSS functional scale (*P* < 0.05), WOMAC function (*P* < 0.05), WOMAC stiffness (*P* < 0.05), and WOMAC total score (*P* < 0.05). In within subjects analyses, all measured outcomes showed improvement at follow-up (*P* < 0.001), except for the borderline distressed group (0.6%) which did not exhibit significant improvements. All ANCOVA analyses were controlled for demographic variables such as age and gender (refer to Tables 3, 4 and 5).

**Table 3:** Becks Depression Scale associated with depressive symptoms among patients with WOMAC Knee Score and Knee Society Score of knee surgery in pre-operative and post-operative settings

Description (*N* = 150)	WOMAC Knee Score	Becks Depression Scale	Knee Society Score	*P* Value
Pre-operative	55.3 ± 4.3	27.2 ± 3.03	32.6 ± 1.68	< .00001
Post-operative (3 months)	43.6 ± 2.57	15.1 ± 1.96	62.6 ± 2.13	< .00001
Post-operative (6 months)	31.4 ± 16.3	7.99 ± 2.12	77.7 ± 2.52	< .00001

**Table 4:** Becks Depression Scale associated with depressive symptoms among male patients with WOMAC Knee Score and Knee Society Score of knee surgery in pre-operative and post-operative settings

Description (*N* = 47)	WOMAC Knee Score	Becks Depression Scale	Knee Society Score	P Value
Pre-operative	55.4 ± 3.38	27.5 ± 3.06	32.2 ± 1.71	< .00001
Post-operative (3 months)	44.06 ± 2.64	15.2 ± 1.91	62.7 ± 2.14	< .00001
Post-operative (6 months)	30.1 ± 3.03	8.38 ± 2.21	77.8 ± 2.63	< .00001

**Table 5:** Becks Depression Scale associated with depressive symptoms among female patients with WOMAC Knee Score and Knee Society Score of knee surgery in pre-operative and post-operative settings

Description (N = 103)	WOMAC Knee Score	Becks Depression Scale	Knee Society Score	*P* Value
Pre-operative	55.3 ± 4.66	27.1 ± 3.02	32.7 ± 1.64	< .00001
Post-operative (3 months)	43.4 ± 2.52	15.04 ± 1.99	62 ± 2.1	< .00001
Post-operative (6 months)	32 ± 19.5	7.81 ± 2.07	77.6 ± 2.48	< .00001

Values are expressed in Mean ± SD

Oneway ANOVA was performed and correlation is significant at *p* < .05

Correlational analyses between range of motion variables and mental health scores were conducted both before and after the surgery, revealing only a few significant correlations, none of which exceeded 0.0001. This implies a minimal relationship between range of motion and psychosocial health scores.

## Regression analysis

Through a stepwise selection process, the inclusion of predictor variables yielded explanations for variations in the follow-up WOMAC function score (13.1%), the follow-up WOMAC stiffness score (10.4%), and the follow-up WOMAC pain score (9.3%)—all follow-up WOMAC variables were subjected to natural log transformation. The final set of retained variables encompassed gender, age, months elapsed since the procedure, Beck's Depression Scale, and the baseline KSS physical functioning score.

Results showed that increased time elapsed since the procedure (*P* < 0.001) corresponded to higher WOMAC scores, as did higher Beck's Depression Scale scores and lower baseline KSS physical functioning scores. This underscores the influence of the passage of time, depression symptoms, and initial physical functionality on predicting outcomes in relation to these WOMAC variables.

Incorporating coexisting conditions alongside age, gender, and baseline physical function did not sufficiently explain the variability in 6-month physical function as measured by WOMAC. However, emotional health continued to be a predictor of 6-month improvement in physical function, even after adjusting for physical coexisting illnesses.

## Discussion

Knee surgery is known to effectively restore physical function and alleviate pain. However, the outcomes of orthopedic knee procedures can vary significantly. Suboptimal functional improvements have been linked to diminished emotional well-being, including symptoms of depression, and inadequate coping mechanisms. In this study, we explored several significant associations between pre-operative depression in patients undergoing total knee arthroplasty. Our primary objective was to examine how pre-operative depressive symptoms serve as a significant and independent predictor affecting various health-related quality of life measures in patients undergoing knee surgery.

Total knee arthroplasty has exhibited remarkable enhancements in the functional capacity, pain resilience, and overall quality of life among individuals afflicted by arthritis. Despite these positive trends, previous research has revealed that the response to arthroplasty benefits is not uniform across all patient groups [24–30]. A small subset of knee arthroplasty recipients express dissatisfaction following replacement surgery [31, 32]. This disparity in outcomes cannot be exclusively attributed to technical glitches or surgical complications; frequently, the underlying cause remains elusive.

Numerous investigations have unearthed a consistent pattern: psychological distress and depression often correlate with unfavorable outcomes, irrespective of the specific surgical procedure [33–35]. This underscores the substantial impact of mental well-being on surgical success and suggests that addressing psychological factors could be a crucial aspect of optimizing post-surgery results [9, 11–18, 33–35].

In our study, a remarkable 99% of patients were initially classified as moderate to severely mentally distressed at baseline, despite not demonstrating heightened limitations in objective disease measures like range of motion. These individuals displayed notably diminished scores across well-being, pain, stiffness, function, and total WOMAC measures. However, although distressed patients have worse baseline measures, their improvement following surgery is significant. Over the course of the study, the prevalence of patients who were classified as moderate to severely depressed decreased from 99% at baseline to 85% who were classified as normal and only 15% patients with mild scores at the 6-month follow-up. These findings suggest that arthroplasty played a significant role in mitigating psychological distress within this subset, likely attributable to the reduction of physical impairment associated with arthritis. Regression analyses was used to predict functional outcomes from arthroplasty. Consistent with prior research, demographic attributes such as gender and age emerged as predictors of these outcomes. Interestingly, the duration of the procedure wielded influence, with briefer interventions correlating with elevated WOMAC scores. Notably, inferior baseline levels of physical function and mental well-being were linked to suboptimal functional progress over the monitoring period. Thus, a multifaceted interplay between mental health and outcomes becomes apparent, where surgical interventions impact mental health markers, and initial pre-operative mental well-being serves as a harbinger of functional recuperation post-surgery [36].

Further exploration is necessary to uncover alternative underlying triggers. Should it be substantiated that addressing mental health issues contributes positively to enhancing surgical outcomes for distressed patients, the feasibility of widely and conveniently administering a concise mental health assessment could emerge. This screening process could effectively flag pre-surgery distressed patients, enabling them to benefit from psychiatric evaluation and potentially therapeutic intervention, both preoperatively and postoperatively, in an effort to enhance emotional well-being and reduce the likelihood of unfavorable outcomes.

Upon analyzing our study findings, it became evident that following surgery, patients exhibited considerable strides in enhancing various outcome metrics, although a handful of constraints were identified. One notable omission was the incorporation of a variable gauging patient expectations pertaining to the procedure. Exploring patient anticipations encompassing procedural intricacies, rehabilitation timelines, and eventual results could potentially shed light on additional outcome variations, thus warranting further investigation. An additional limitation shared with studies evaluating subjective indicators such as overall well-being, social integration, pain perception, and physical rigidity also emerged. Despite our utilization of standardized tools validated within pertinent populations, the inherent subjectivity tied to patient testimonials could introduce inherent biases in the subjective measurements.

## Conclusion

Our study found that following surgery, depressed patients had appreciable rates of improvement on most outcome measures. The results of this study indicate that the relationship between mental health and outcome of the surgery is bidirectional, with surgery leading to significant improvements in mental health status, whereas worse pre-operative mental health status is a predictor of relatively worse outcomes from surgery. We believe that the results of this study, and the possible evaluation and treatment of mental health preoperatively with simple tools in clinical practice may have a direct positive impact on surgical outcomes as well as improving mental health concerns of these patients.

### Supplementary Information

**Additional file 1.** A cohort flow diagram showing number of patients who met inclusion criteria and were assessed with various scoring methods.
