# Supplementary material for: Surgical healing beyond the scalpel: exploring the impact of depressive symptoms on functional recovery in total knee arthroplasty patients
Source: J Orthop Surg Res. 2023 Nov 4;18:833. doi: 10.1186/s13018-023-04302-6 (PMC10625223; doi:10.1186/s13018-023-04302-6)
Supplement: Supplementary file 1 — Additional file 1. A cohort flow diagram showing number of patients who met inclusion criteria and were assessed with various scoring methods. [file 13018_2023_4302_MOESM1_ESM.docx]

**Flow Diagram**

## Analysis

## Follow-Up

WOMAC Score

## Assessed

Becks Depression Scale

KSS Score

Included (n= 150)

Excluded (n=21)

♦  Not meeting inclusion criteria (n=10)

♦  Declined to participate (n=5)

♦  Other reasons (n=6)

Assessed for eligibility (n=171)
